# 4470 Are nurses’ attitudes toward caring for hospitalized adults with intellectual disabilities associated with nurse and nursing unit characteristics?

**DOI:** 10.1017/cts.2020.202

**Published:** 2020-07-29

**Authors:** Melissa Lynn Desroches

**Affiliations:** 1 Tufts University CTSI

## Abstract

OBJECTIVES/GOALS: (1) Determine nurse (age, education level, years of experience, ID education/training, contact with people with ID, communication apprehension, beliefs about patient quality of life), and nursing unit (teamwork, staffing and resources, person-centered care) characteristics that are associated with and predictive of nurses’ attitudes, positive emotions, and negative emotions toward caring for adults with ID. (2) Explore nurses’ perspectives of perceived barriers and facilitators to providing nursing care to hospitalized persons with ID and medical comorbidity, and how nursing care differs when caring for a person with ID. METHODS/STUDY POPULATION: This mixed methods nested analysis will employ an internet survey of medical-surgical registered nurses to collect nurses’ attitudes and emotions toward caring for hospitalized persons with ID and medical comorbidity, nurse characteristics, and nursing unit characteristics. We intend to recruit 150 medical surgical nurses currently practicing in the United States via email invitation to the membership of the Academy of Medical Surgical Nurses. Purposeful maximum variation sampling will be used to invite a subset of respondents for qualitative, semi-structured telephone interviews to elicit barriers and facilitators to nursing care of persons with ID and how nursing care differs when caring for persons with ID. RESULTS/ANTICIPATED RESULTS: We hypothesize that lower nurse education level, fewer years of experience, less ID education/training, lower amount of contact with people with ID, increased communication apprehension, and lower beliefs about the quality of life of persons with ID will be associated with negative nurse attitudes and emotions toward caring for people with ID. Further, we hypothesize that lower levels of nursing unit teamwork, staffing and resources, and person-centered care practices will be associated with negative nurse attitudes and emotions toward caring for people with ID. DISCUSSION/SIGNIFICANCE OF IMPACT: The proposed research is an important first step in determining potential nurse and nursing unit factors influencing nurses’ attitudes toward caring for people with ID. It will lead to targeted interventions to enhance nursing care quality and reduce hospital-associated healthcare disparities among people hospitalized with intellectual disabilities and medical comorbidities.

